# Distribution of the Primary Endosymbiont (*Candidatus* Uzinura Diaspidicola) Within Host Insects from the Scale Insect Family Diaspididae

**DOI:** 10.3390/insects3010262

**Published:** 2012-02-29

**Authors:** Matthew E. Gruwell, Meghan Flarhety, Katharina Dittmar

**Affiliations:** 1 Penn State Erie, School of Science. P-1 Prischak Building, 4205 College Drive, Erie, PA 16563, USA; E-Mail: mef5089@psu.edu; 2 Department of Biological Sciences, 109 Cooke Hall, SUNY at Buffalo, Buffalo, NY 14260, USA; E-Mail: katharinad@gmail.com

**Keywords:** *in-situ* hybridization, endosymbiont, *Uzinura*

## Abstract

It has long been known that armored scale insects harbor endosymbiotic bacteria inside specialized cells called bacteriocytes. Originally, these endosymbionts were thought to be fungal symbionts but they are now known to be bacterial and have been named *Uzinura diaspidicola*. Bacteriocyte and endosymbiont distribution patterns within host insects were visualized using *in situ* hybridization via 16S rRNA specific probes. Images of scale insect embryos, eggs and adult scale insects show patterns of localized bacteriocytes in embryos and randomly distributed bacteriocytes in adults. The symbiont pocket was not found in the armored scale insect eggs that were tested. The pattern of dispersed bacteriocytes in adult scale insects suggest that *Uzinura* and *Blattabacteria* may share some homologous traits that coincide with similar life style requirements, such as dispersal in fat bodies and uric acid recycling.

## 1. Introduction

Scale insects (Coccoidea) are important plant pests that are generally sedentary, even immobile at times. They feed exclusively on plants, often causing serious agricultural damage [1], and are most commonly encountered by growers and plant enthusiasts as soft scales (Coccidae) and mealybugs (Pseudococcidae). The most diverse lineage of scale insects is the Diaspididiae, also known as armored or hard scale insects because of their waxy covering that completely hides the immobile insect while it grows and feeds. The armored scale insects are comprised of over 2400 species [2] that primarily feed on woody plants and grasses. They exhibit extreme sexual dimorphism in that adult females are immobile, eyeless, wingless, legless, and sac-like without distinction between body segments [3]. Males possess the typical appendages and actively fly around seeking opportunities to mate with females [3]. Males are also genetically haploid via paternal genome elimination and females are diploid, however males are often absent making some lineages parthenogenetic [4]. There are about 200 species of armored scale insects that are considered serious economic pests [5] and some species such as *Apsidiotus nerii* are extreme generalists known to feed on over 100 families of plants [6].

As is the case with many other plant feeding insects, the scale insect diet lacks many essential nutrients. So far, a few scale insect groups have known obligate (or primary) bacterial endosymbionts such as *Tremblaya phenacola*, *Tremblaya princeps, Moranella endobia* (an obligate known to live within *Tremblaya princeps*), *Brownia rhizoecola* in mealybugs [7,8] and *Uzinura diaspidicola* in armored scale insects [9]. Endosymbiont names are normally designated with the formal label *Candidatus* to denote the inability to grow such bacteria in culture, followed by the genus and species name. In this publication we will follow convention and refer to symbiotic bacteria by their informal genus and species names, lacking *Candidatus*. Though these endosymbionts are found in closely related insects, the endosymbionts are often not closely related bacteria. For instance, *Tremblaya* is in the β-Proteobacteria and *Brownia* and *Uzinura* are part of the Bacteroidetes. There is other evidence of probable endosymbionts in scale insects, such as Bacteroidetes in *Rastrococcus*, *Cryptococcus* and Iceryini [8]; and γ-Proteobacteria in Coccidae [10]. Endosymbiont diversity within mealybugs, and between scale insect families suggests a large diversity of obligate endosymbionts may be present in the Coccoidea, however, endosymbiosis in most scale insect families is currently unexplored.

Though armored scale insect endosymbionts were only recently studied molecularly, patterns akin to endosymbiosis were originally described in 1866 by Metschnikoff [11] and in 1910, Sulc interpreted the images as symbiotic organisms within the insects [12]. Early investigators such as Buchner [13] described diaspidid obligate endosymbionts as fungi while Tremblay had correctly hypothesized that they appeared to be bacteria, which was later confirmed [14,15,16]. Buchner’s research provided insights into the 5n pentaploid cells (termed mycetocytes by Buchner, now called bacteriocytes) and the inconsistency of endosymbiont numbers between adult males (few) and adult females (many), which is expected in a system where males do not feed and females are laying eggs. Tremblay [16] provided size and shape descriptions as endosymbionts change from oval (1–2 μm long) to ramified bodies (~9 μm long). Endosymbionts are passed directly from mother to offspring as either individual bacteria, or within bacteriocytes, depending upon the host species [16]. Bacteriocytes are not known to form a specific bacteriome as is found in mealybugs and aphids [13,15,17] but are dispersed throughout the cell. 

As with other plant and blood feeding insects, there is renewed interest in armored scale insect/endosymbiont systems. *Uzinura* has been shown to follow a strict vertical inheritance pattern from mother to offspring, lacking evidence of horizontal transfer between species of host scale insects [9]. *Uzinura* is closely related to *Blattabacteria*, the primary endosymbiont of cockroaches, and *Sulcia* an obligate endosymbiont of many Auchenorrhynchan insects. Along with other evidence, both of these endosymbionts are shown to be obligate because they have reduced genomes and an estimated 13% and 20% of their genes are used in amino acid metabolism, respectively [18,19,20]. *Sulcia* endosymbionts are localized in a bacteriome, but *Blattabacteria* are not. Here we provide *in-situ* hybridization images of the obligate endosymbiont *Uzinura* in various armored scale insect species to visualize the pattern of symbiont distribution and better understand correlations with similar endosymbionts.

## 2. Experimental Section

### 2.1. Collection and Taxonomy

Images of *Uzinura diaspidicola* were taken from the armored scale insect species *Diaspis echinocacti*, *Hemiberlesia lataniea*, and, *Chionaspis pinifoliae* and additional data was taken from similar protocols done on *Pseudalacaspis sp.* and *Chrysomphalus dictyospermi*. The images depict bacteriocyte arrangements in the major life stages of armored scale insects, including the embryo, egg and adult. Image 1A is from an adult female, *D. echinocacti*. Image 1B is as an embryo from *D. echinocacti* that was displaced outside of the adult female during the process of slide mounting. Image 1C is an egg from *C. pinifoliae*. Images 1D and 1E are adult females from the species *H. lataniae.* Over twenty additional slides of all life stages and from all species mentioned were inspected and used for data collection.

All samples except *C. pinifoliae* were collected through cooperation with the Buffalo Botanical Gardens, Buffalo NY. *C. pinifoliae* was collected on the SUNY at Buffalo campus (49°59'59.46"N, 78°47'24.97"W). 

### 2.2. Incubation and Slide Mounting

Live insects were carefully removed from plant tissues, and pierced using a size 0 insect pin allowing for absorption of solutions. Scale insect samples were placed in 1.5 ml centrifuge tubes containing 200 μL Carnoy’s solution at room temperature for three days. Carnoy’s was removed and scales were then immersed in 200μl of 10% H_2_O_2_ at room temperature for 3 days. H_2_O_2_ was replaced with 100 μL of 2X hybridization solution [17] along with 3 μL of UZI319 probe created after a small portion of the 16s rRNA gene, TGGTCCGTATCTCAGTAC. The probe was created following the CF319A probe originally designed for *Sulcia* by Moran *et al.* [21] which was designed to start from the 319^th^ base of Domain I of the 16s rRNA gene, TGGTCCGTGTCTCAGTAC. Once the hybridization solution and the probe were added, the centrifuge tube was covered with aluminum foil to block all light and incubated for 24 hours in a water bath at 50 °C. 

The remaining steps were done under red light to not destroy the effectiveness of the probe. The hybridization solution with probe was removed and the scale insects were rinsed with 100 μL of 1X SSC for 10 minutes followed by another rinse of the same solution for 2 minutes. The scale insects were then incubated in 100 μL PBS solution in preparation for slide making. 

### 2.3. Mounting of Slides

Treated insects were placed in a 40 μL drop of 15% Glycerol in PBS solution on the middle of the slide and inspected for folding and orientation. A coverslip was placed over the insects and a thin layer of clear nail polish was applied around the perimeter of the coverslip to avoid desiccation and stored in the dark until inspection. Slides were inspected on a Zeiss Axioimager Confocal microscope in the SUNY Buffalo microscopy center and a Leica TCS sp5 confocal microscope at Penn State Behrend School of Science.

## 3. Results and Discussion

**Figure 1:**
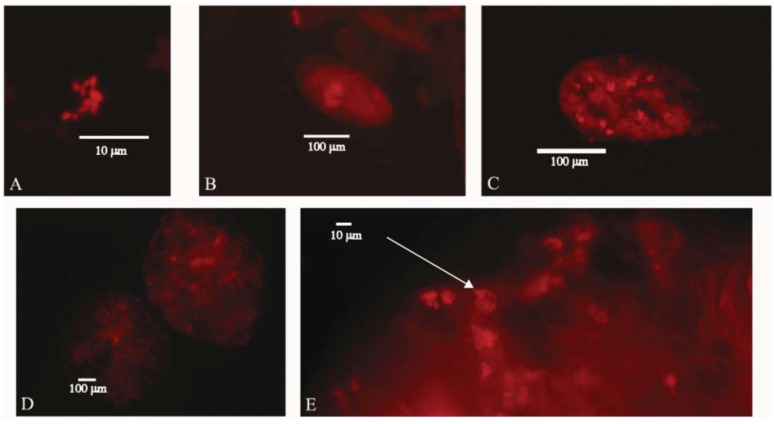
*In situ* hybridization images of the bacterial endosymbiont *Uzinura diaspidicola* from various armored scale insect species. Image (**A**) Individual bacteria from *Diaspis echinocacti*, (**B**) *Diaspis echinocacti* embryo (**C**) Single *Chionaspis pinifoliae* egg from. (**D**) Two whole *Hemiberlesia lataniae* adult female insects (**E**) Partial *Hemiberlesia lataniae* adult showing internal embryo and arrow denoting symbiont pocket in an single embryo.

The resulting hybridization images of *Uzinura diaspidicola* demonstrate endosymbiont distribution patterns unique among scale insects. It had been previously described by Tremblay [16] that adult armored scale insect bacteriocytes do not form a distinct organ or bacteriome as in other insects; rather the bacteriocytes are randomly distributed throughout the organism. Image 1D confirms prior research and shows the lack of a bacteriome as bacteriocytes are dispersed throughout the adult organism. Conversely, Tremblay [16] drew images of bacteria clustering in one pole to form a “symbiont pocket” at early embryonic stages and eggs. Our images of the endosymbionts confirm this result in the embryos showing the symbiont pocket from image 1E. However, image 1C depicting the egg close up demonstrates random dispersal of bacteriocytes contrary to Tremblay [15] (Figure 1). This may be the result of different ages of the internal embryos and external eggs that were studied, or because the embryos and eggs were visualized from different species. Tremblay used images from *Pseudalacaspis pentagona* in his experiments but we were unable to find eggs associated with our *Pseudalacaspis* samples. 

According to the images of *Uzinura diaspidicola*, the bacteria are slightly oblong in shape and average 2–3 μm in length (Figure 1A). Bacteriocytes range in size between 10–20 μm. Individual embryos range in number of bacteriocytes between 3 (Figure 1B) and 13 (Figure 1D) though bacteriocytes within embryos residing inside the adult scale are difficult to quantify exactly due to the possibility that bacteriocytes are actually in the adult scale rather than the embryo. Having obtained data from over 20 individuals we have determined that adult female scale insects average 122–165 bacteriocytes per individual, though this number may include a small number of embryonic bacteriocytes due to reasons outlined above.

Evidence for the importance of endosymbiosis in the evolution of plant feeding insects continues to increase in many groups that can be considered model systems for this phenomenon such as *Buchnera* in aphids, *Blattabacteria* in cockroaches, *Sulcia and Baumannia* in Auchenorrynchan insects and *Carsonella* in psyllids [18,19,22,23,24,25]. As genomic data becomes available we are finding that obligate endosymbionts have enabled a lifestyle of feeding on nutritionally poor plant sap and therefore are potentially driving coevolution with plants. *Uzinura* endosymbionts, though still in the early stages of scientific exploration, appear to be just as important in evolution of plant insect interactions and diversity in armored scale insects. However, key differences in the *Uzinura*/armored scale insect system seem to influence bacteriocyte patterns.

These differences are the feeding mechanisms and potential use of waste within the host insects. Armored scale insects are oddities in that they do not excrete honeydew, a trait found in nearly all other sternorrynchan insects. It is still unknown exactly how they feed, but lacking honeydew production limits them to low pressure tissues and at least some are known to feed on parenchyma rather than plant sap [26]. Further, they demonstrate no excretion of waste and they lack a complete gut [27]. This leads to questions about what they are doing with waste products and how that might be correlated with distribution patterns of endosymbionts. The answer to such inquires may lie in the close relative of *Uzinura*, the cockroach endosymbiont *Blattabacteria*.

The American cockroach *Periplaneta americana* do not excrete uric acid, a substance common in insect frass that is excreted as a function of purging excess nitrogen from the insect [28]. Even when cockroaches were force fed large amounts of protein nitrogen, urate was only detected in frass when protein nitrate consisted of over 1% of the food weight, and in this case it was likely detected because traces of it passed through the gut unabsorbed [29]. When these cockroaches were given antibiotics to kill endosymbionts, fat bodies formerly holding endosymbionts showed a 20 fold increase of uric acid, demonstrating the likelihood of uric acid recycling by endosymbiotic bacteria *Blattabacteria* [30]. Other cockroaches are known to store urate in the fat body as well [31]. Recent genomic work done on *Blattabacteria* shows that this bacterium has the necessary metabolic genes to recycle nitrogen from the byproducts of uric acid degradation, which are urea and ammonia [18]. Further, these endosymbionts contain the genes for making all but four amino acids from the products of uric acid break down and a few other sources [18]. These bacteria are found in bacteriocytes within the fat bodies of cockroaches, presumably to be more efficient in nitrogen recycling, as this is where the cockroaches store uric acid crystals. 

## 4. Conclusions

Considering possible similar endosymbiotic needs between the armored scale insects and cockroaches, such as endosymbiontic distribution throughout the body (associated with fat body in cockroaches and likely associated with fat bodies scales though this has not been shown), and not excreting nitrogenous waste, we suggest that *Uzinura* may provide benefits to scale insects that are similar to those provided to cockroaches by *Blattabacteria*. This would potentially explain the oddities of the armored scale insects/*Uzinura* relationship such as the lack of a bacteriome and random bacteriocyte distribution that we have found and gives clues toward a better understanding of feeding habits and other metabolic pathways that are occurring within the insect. Sequencing the genome of *Uzinura* would be the next logical step toward confirming this hypothesis specifically if we find genes similar to those of *Blattabacteria*, namely genes for amino acid synthesis from uric acid products [18]. Additional experiments to better understand food sources and waste storage in armored scales will improve our knowledge of the host / endosymbiont interaction in these insects.
